# ATM-dependent phosphorylation of SNEV^hPrp19/hPso4^ is involved in extending cellular life span and suppression of apoptosis

**DOI:** 10.18632/aging.100452

**Published:** 2012-04-20

**Authors:** Hanna Dellago, Abdulhameed Khan, Monika Nussbacher, Anna Gstraunthaler, Ingo Lämmermann, Markus Schosserer, Christoph Mück, Dorothea Anrather, Annika Scheffold, Gustav Ammerer, Pidder Jansen-Dürr, Karl Lenhard Rudolph, Regina Voglauer-Grillari, Johannes Grillari

**Affiliations:** ^1^ Department of Biotechnology, University of Natural Resources and Life Sciences Vienna, Vienna, Austria; ^2^ Institute of Biomedical Aging Research, Austrian Academy of Sciences, Innsbruck, Austria; ^3^ Institute of Biomedical Aging Research, Austrian Academy of Sciences, Innsbruck, Austria; ^4^ Institute of Molecular Medicine and Max-Planck-Research Group on Stem Cell Aging, University of Ulm, Germany; ^5^ Evercyte GmbH, Muthgasse 18, 1190 Vienna, Austria

**Keywords:** SNEV, Prp19, Pso4, ATM, oxidative stress, endothelial cells, replicative senescence

## Abstract

Defective DNA repair is widely acknowledged to negatively impact on healthy aging, since mutations in DNA repair factors lead to accelerated and premature aging. However, the opposite, namely if improved DNA repair will also increase the life or health span is less clear, and only few studies have tested if overexpression of DNA repair factors modulates life and health span in cells or organisms. Recently, we identified and characterized SNEV^hPrp19/hPso4^, a protein that plays a role in DNA repair and pre-mRNA splicing, and observed a doubling of the replicative life span upon ectopic overexpression, accompanied by lower basal DNA damage and apoptosis levels as well as an increased resistance to oxidative stress. Here we find that SNEV^hPrp19/hPso4^ is phosphorylated at S149 in an ataxia telangiectasia mutated protein (ATM)-dependent manner in response to oxidative stress and DNA double strand break inducing agents. By overexpressing wild-type SNEV^hPrp19/hPso4^ and a phosphorylation-deficient point-mutant, we found that S149 phosphorylation is necessary for mediating the resistance to apoptosis upon oxidative stress and is partially necessary for elongating the cellular life span. Therefore, ATM dependent phosphorylation of SNEV^hPrp19/hPso4^ upon DNA damage or oxidative stress might represent a novel axis capable of modulating cellular life span.

## INTRODUCTION

Accumulation of DNA damage, if not repaired, can lead to premature aging, as indicated by several inherited diseases caused by mutations of DNA damage response factors that show features of premature aging [1], [2]. Furthermore, increased exposure to DNA damage might lead to the development of premature aging characteristics, e.g. in long-term survivors of chemotherapy, for which we proposed to use the term acquired premature progeroid syndrome (APPS; [3]).

One of the proteins orchestrating DNA damage response is ATM (ataxia telangiectasia mutated), which plays a central and multiple role in the cellular stress response by monitoring and maintaining DNA integrity (reviewed by [4]). These crucial functions are mirrored by the disorder caused by its mutations, Ataxia telangiectasia (A-T), which is also classified as a segmental progeroid syndrome [5] and includes symptoms like cerebellar degeneration, immuno-deficency, genomic instability, thymic and gonadal atrophy and cancer predisposition [6].

Biochemically, one of these functions is control of the cell cycle in response to DNA damage. This is exerted by ATM-dependent induction of p53 that leads to activation of the CDK inhibitor p21, resulting in inhibition of the Cyclin-E/CDK2 complex and inhibition of progression from G1 into S-phase [7].as well as a CHK2 phsophorylation dependent G2/M arrest [8].

An equally important function of ATM is in effectively repairing DNA double-strand breaks. Upon DNA damage, ATM is auto-phosphorylated leading to dissociation of the inactive dimer to active monomers, which are rapidly recruited to DNA DSB sites together with the MRN (Mre11, Rad50, NBS1) complex (reviewed by [4]. Then, ATM phosphorylates the histone variant H2AX to γH2AX, which provides a docking station for many repair factors and signal transducers like p53 and BRCA1, which are in turn phosphorylated by ATM [9,10]. In addition, ATM activity on DNA DSB sites enables the relaxation of heterochromatin, which facilitates access of repair proteins to the damaged DNA [11].

A more recently identified function of ATM has been observed in the response to oxidative stress [12], probably by sensing reactive oxygen species (ROS) [13]. ROS impact on signal transduction, aging/senescence, apoptosis, neuromodulation and antioxidant gene modulation, in addition atherosclerosis and several neurodegenerative diseases [14], and at least part of these effects might involve ATM, as oxidative stress response pathways are constitutively active in A-T cells [13,15].

Finally, a link between ATM and the pentose phosphate pathway is established (PPP, reviewed in [16]. After DNA damage, ATM stimulates PPP in order to generate nucleotides and NADPH needed for DNA repair. NADPH thereby not only provides the reducing equivalents for biosynthetic reactions but also for regeneration of the major ROS scavenger glutathione [17].

Recently, a consensus phosphorylation motif recognized by ATM and ATR (ATM and Rad3-related; a kinase that shares many substrates with ATM, but responds to distinct types of DNA damage) was identified [18].

To our surprise, the published consensus site is present in the amino acid sequence of SNEV^hPrp19/hPso4^ (for simplicity reasons termed SNEV in the following), a “multi-talented” protein involved in pre-mRNA splicing [19-22], DNA repair [23-25], senescence and extension of life span [26,27] and neuronal differentiation [28,29]. SNEV increases replicative resistance to oxidative stress, reduces basal levels of DNA damage and apoptosis and increases cellular life span in vitro [26]. The role of SNEV in the mammalian DNA damage response (DDR) first emerged with the report that it was strongly upregulated by DNA damage in human cells, and that its depletion by siRNA resulted in an accumulation of double-strand breaks (DSBs), apoptosis and reduced survival after exposure to ionizing radiation [23]. Subsequently, Zhang and collaborators (2005) have shown that Cdc5L, a member of the SNEV core complex together with PLRG1 and SPF27/BCAS2, directly interacted with the Werner syndrome protein (WRN) and is involved in early steps of DNA interstrand cross link (ICL) repair. WRN is a DNA helicase that has roles in homologous recombination and the processing of stalled replication forks in response to DNA damage. Mutations of WRN are the cause of Werner's syndrome, one of the segmental progeroid syndromes that most strikingly resemble aging (reviewed by [30]. DNA damage also induces the attachment of ubiquitin to a subfraction of endogenous SNEV via a thiolester linkage, and this modification disrupts the interaction between SNEV and both Cdc5L and PLRG1 demonstrating that DNA damage can profoundly affect the structure of the SNEV core complex [24]. Recently, the SNEV complex has been connected to DNA damage checkpoint signaling pathways. Cdc5L interacts physically with the cell-cycle checkpoint kinase ataxia-telangiectasia and Rad3-related (ATR). Depletion of Cdc5L by RNA-mediated interference methods results in a defective S-phase cell-cycle checkpoint and cellular sensitivity in response to replication-fork blocking agents [31].

While mutations and deletions of several DNA repair factors are known to cause progeroid syndromes, there is very little known on the effects of overexpressing DNA repair factors on the life spans of model systems. As SNEV is known to extend the replicative life span of normal human cells, it would be of some interest to see if this effect is due to its DNA repair function.

Here, we show that indeed SNEVis phosphorylated in response to oxidative stress and different DNA damaging agents, and that phosphoSNEV is located predominantly in the nucleus. Moreover, we examined the impact of the phosphorylation on repair capacity and life span in cultured human cells by overexoressing wt and phosphorylation incompetent SNEVand show that phosphorylation at S149 is essential for mediating the cytoprotective effect of SNEVupon DNA damage/oxidative stress and partially contributes to the life span extension caused by SNEV overexpression.

## RESULTS

### Oxidative stress induces an additional SNEV protein band in Western blotting

**Figure 1:**
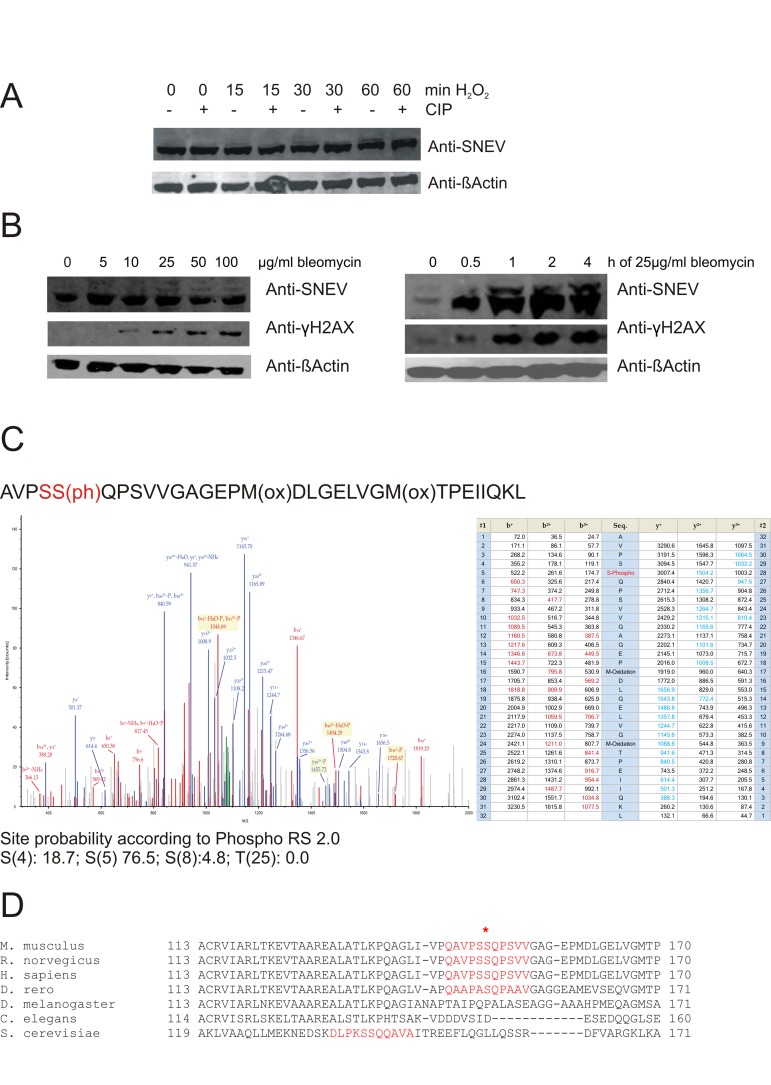
Upon oxidative stress, SNEV is detected as double band, probably representing a phosphorylated species (**A**) Upon treatment with hydrogen peroxide, additional bands of higher molecular weight were detected with anti-SNEV antibody. These bands disappear upon incubation with phosphatase. (**B**) Similarly, upon bleomycin treatment of fibroblasts, we detected an additional band with anti-SNEV antibody, which increased in a dose-and time-dpendent manner. Fibrolblasts were incubated with 0, 5, 10, 25, 50 or 100μg/ml bleomycin for 1hour (left panels) or with 25μg/ml bleomycin for 0, 0.5, 1, 2, 4 hours (right panels), scraped on ice in 2x SDS loading dye and subjected to Western Blotting with anti-SNEV antibody. Anti-β-actin was used to ensure equal loading. Anti-γH2AX antibody was used to confirm that the treatment induces DNA damage. (**C**)Collision-induced dissociation spectrum of the SNEV peptide AVPSS(ph)QPSVVGAGEPM(ox)DLG Indeed, a phosphorylation was detected and assigned to S149 with a probability of 76.5% (Fig. 1C). While the consistence of three different spectra with different m/z ratios underscores the correct assignment of the phosphorylation to this site, the remaining uncertainty comes from the presence of 3 serine residues within a quite long peptide. Together with the problem of lack of trypsin or chymotrypsin proteolytic sites near these serines, a better proof of S149 as the really phosphorylated serine by mass spectrometry is hampered. (**D**) Sequence comparison of SNEV homologues in the putative ATM target site. The consensus sequence surrounding the phospho-SQ site on ATM substrates that are regulated by DNA damage is conserved in the SNEVamino acid sequences across different vertebrates, but not in non-vertebrates.

From previous results we know that SNEVexpression is induced upon oxidative and DNA DSB damage [23,26]. To further study the effect of oxidative stress on SNEV, we treated HeLa cells with 1mM hydrogen peroxide for different times and detected additional bands above the expected 56 kDa band in a Western Blot with anti-SNEV antibody, and band intensity increased in a time-dependent manner. These higher bands were removed after dephosphorylation using calf intestinal phosphatase (Fig. 1A). Similarly, this additional SNEVband appeared when we treated human diploid fibroblasts (HDF5) with bleomycin (Fig. 1B).

These results prompted us to ask if these bands might represent a phosphorylated form of SNEV in response to oxidative stress. Therefore, we isolated SNEV as GFP-fusion from stable HeLa cells constitutively expressing GFP-tagged SNEVunder the control of the endogenous promoter as described in [32] and submitted it to mass spectrometric analysis. The advantage of using GFP-SNEV-Hela lies in allowing the use of a commercially available GFP-antibody for IP which is covalently coupled to agarose beads, thus avoiding predominant detection of immunglobulins in the mass spectrometric analysis. In order to make sure that GFP-SNEV behaves similarly to SNEV in cells we confirmed its localization to nuclei (Fig. S1A) as well as its ability to integrate into the SNEV/CDC5L complex by co-precipitation of SNEV and CDC5L with GFP-SNEV by GFP antibodies (Fig. S1B).

Interestingly, S149 also represents an ATM target site (Fig. 1D). Fig. S2 shows that the signal detected by MS is specific for H_2_O_2_-treated cells. This is not due to a difference in the efficiency of the IP, since the Western Blot in fig. S1B clearly shows that comparable amounts of GFP-SNEV were precipitated under both conditions. Table S1 gives a list of the identified phosphopeptides of SNEV in the H_2_O_2_-treated sample with site probability calculated by phosphoRS 2.0.

Taken together, the mass spec spectra, the presence of an ATM kinase consensus site, and ATM's known function as a kinase signaling oxidative stress prompted us to generate an anti-pSNEV(S149) specific antibody.

### Phosphorylation of SNEV at S149 is induced by oxidative stress in different cell types

**Figure 2:**
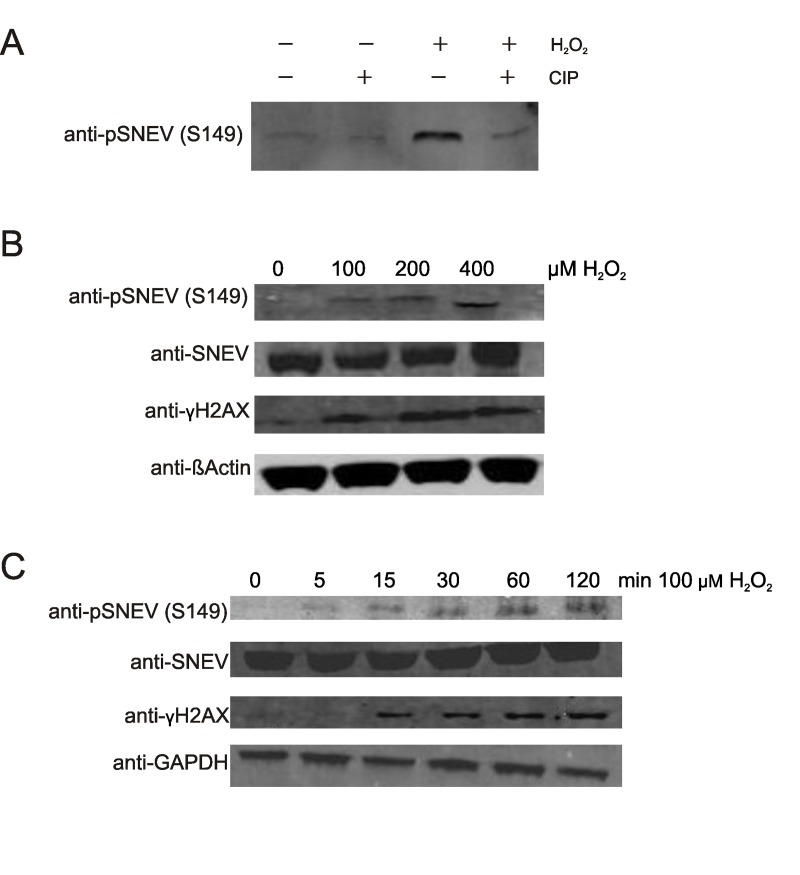
Phosphorylation of SNEV at serine 149 is induced by oxidative stress in a time- and dose dependent manner (**A**) Upon oxidative stress treatment, the band detected by a phospho-SNEVspecific antibody (anti-pSNEV(S149)) increases, and diminishes upon phosphatase treatment. HeLa cells were treated with 1mM H_2_O_2_ for 1h or left untreated. Lysates were incubated with CIP and subjected to Western Blotting with a specific anti-phosphoSNEV(S149) antibody, that was generated by immunizing rabbits with an artificial SNEV-phosphopeptide. (**B**) Human diploid fibroblasts (HDF) were treated with 0, 100, 200 or 400 μM H_2_O_2_ for 1h, scraped on ice in 2x SDS loading dye and subjected to SDS PAGE. Western Blot was detected with anti-pSNEV(S149) and anti-SNEV to compare pSNEV to total SNEV levels. anti-γH2AX antibody was used as positive control for stress-dependent phosphorylation. anti-?-Actin was used as loading control. (**C**) HDF were treated with 100 μM H_2_O_2_ for 0, 5, 15, 30, 60 or 120 min. Lysis and Western Blot as in **B**.

In order to take a closer look at this possible phosphorylation site of SNEV, a phospho-specific antibody was generated. This anti-pSNEV(S149) antibody detects a band at the expected size of SNEVupon oxidative stress treatment of Hela cells, and this signal is abolished upon phosphatase treatment (Fig. 2A). Besides the bands at the expected size, other bands are as well detected by anti-pSNEV(S149) antibody on Western Blot of Hela lysates (Fig. S3), but at higher molecular weights. These bands might represent post-translationally modified SNEV in complexes similar to the ones described in [24].

Based on these results, we were curious to confirm our assumption that SNEV phosphorylation was stimulated by DNA damage in normal cells. Human diploid fibroblasts (HDF5) were exposed to different concentrations of hydrogen peroxide for 1 hour or to 100μM hydrogen peroxide for different times. Total cell lysates were subjected to Western Blot analysis with anti-pSNEV(S149). Indeed, phosphorylation of SNEV was triggered by oxidative stress in a time- and dose-dependent manner (Fig. 2B and C). Phosphorylation was observed as quickly as after 5 minutes of hydrogen peroxide treatment and reached a maximum after 1 hour. To verify that hydrogen peroxide treatment indeed induced the formation of DNA double strand breaks (DSB), we examined the ATM dependent phosphorylation of γH2AX, which occurs in response to DSB [7], and showed a similar kinetic as phosphorylation of SNEV(Fig. 2B and C).

### Different types of DNA damage induce nuclear localization of pSNEV

In order to localize pSNEV within the cells, we first tested for the specificity of the antibody for indirect immunofluorescence studies. Using either the phospho-peptide that was used for generation of the antibody or a non-phospho-peptide with the same sequence, for pre-blocking the anti-pSNEV antibody, staining was abolished by blocking with the phospho-peptide, while no difference was seen after pre-incubation with the non-phospho-peptide (Fig. S4).

**Figure 3:**
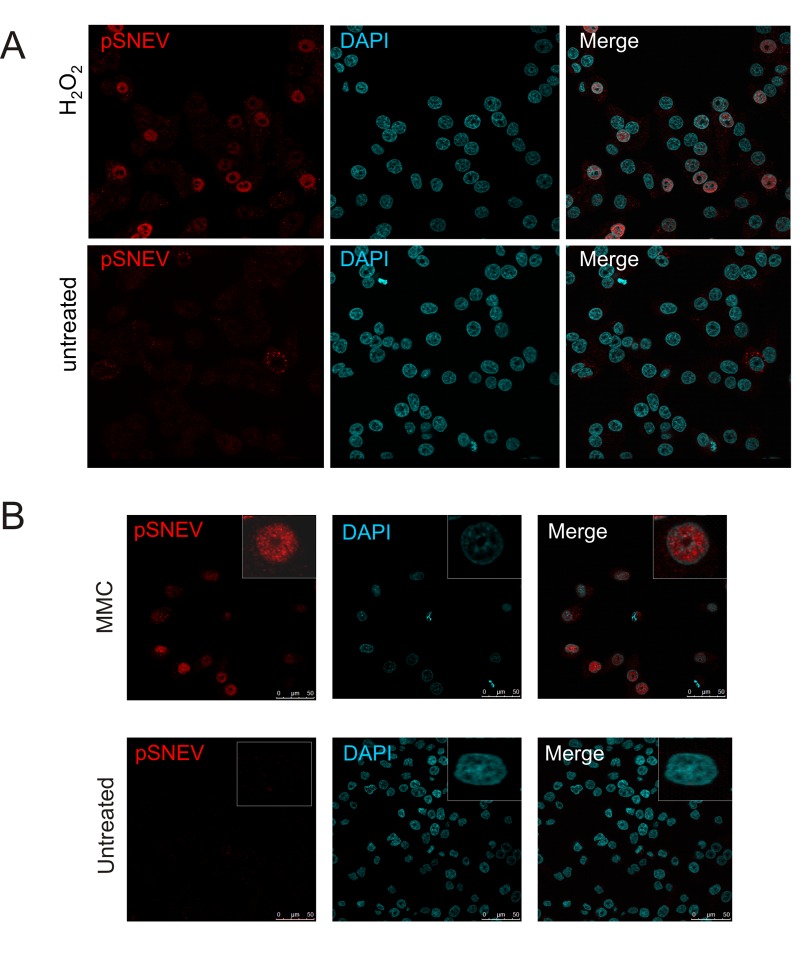
Upon oxidative stress, SNEV is phosphorylated and localizes mainly to the nucleus (**A**) Hela cells were seeded on coverslipsand treated with 100 μMH_2_O_2_ for 1 hour. Cells were stained with anti-pSNEV(S149) and DAPI as described in the Materials and Methods section and subjected to fluorescence microscopy. (**B**) HeLa cells were treated with 25 μg/ml MMC for 24 hours prior to indirect immunofluorescence staining with anti-pSNEV(S149) antibody.

In addition to a strong and predominant nuclear signal upon oxidative stress, the phosphorylated form is also detected in the cytoplasm (Fig, 3A), and it is currently unclear if this phosphorylation is a signal for shuttling of SNEV between nucleus and cytoplasm. In any case, under normal conditions only very few cells stained positive for phosphoSNEV, which seems to localize to the cytoplasm (Fig. 3A).

To test if phosphorylation of SNEVis restricted to oxidative stress conditions or if it is a general step in the DNA damage response, we challenged cells with mitomycin C (MMC), a potent DNA crosslinker. HeLa cells were incubated with 25μg/ml MMC for 24 hours and submitted to indirect immunofluorescence staining with anti-pSNEV(S149). As observed for hydrogen proxide, also MMC induces phosphorylation of SNEV, and the phosphorylated species is mainly detected in the nucleus (Fig. 3B).

Thus, we assume that the phosphorylation of SNEVis an integral part of the nuclear DNA damage response.

### Phosphorylation of SNEV at serine 149 is ATM-dependent

In order to test if this phosphorylation is dependent on ATM, we took advantage of the reagent 2-(4-Morpholinyl)-6-(1-thianthrenyl)-4*H*-pyran-4-one (KU-55933), a specific inhibitor of ATM kinase activity [33].

**Figure 4:**
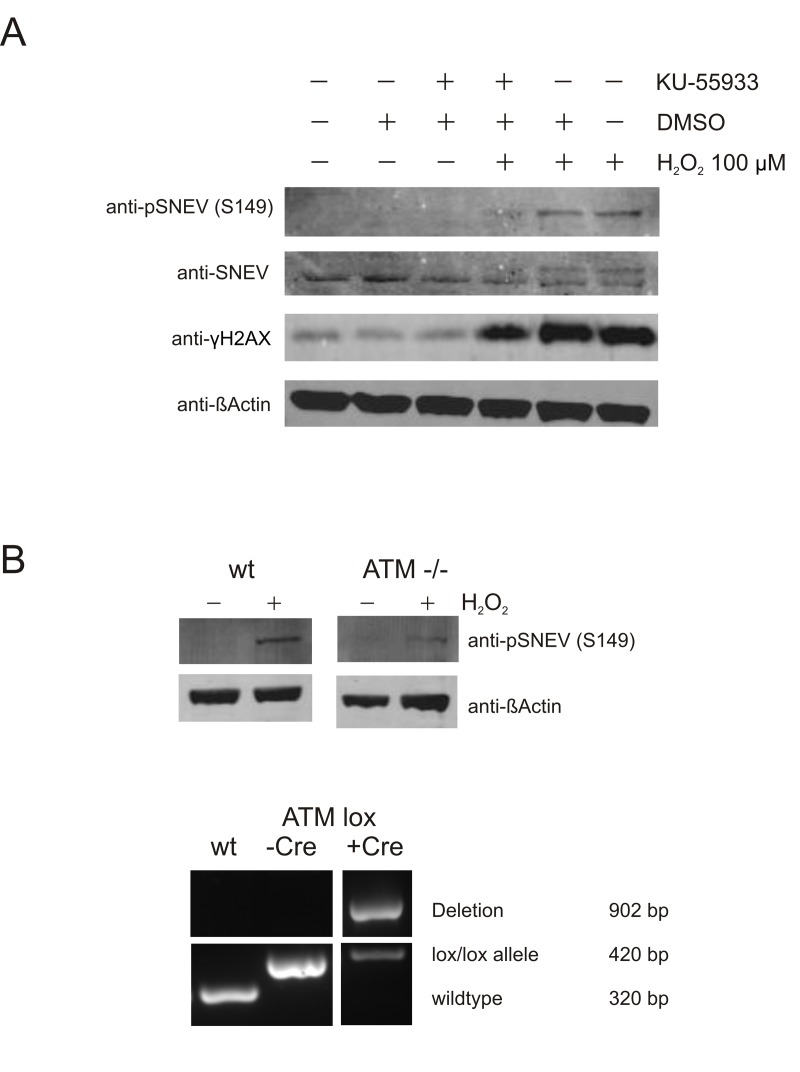
Phosphorylation of SNEV at S149A is ATM-dependent (**A**) ATM inhibition alleviates phosphorylation of SNEV in response to oxidative stress. HDFs were treated with 100 μM H_2_O_2_ and the specific ATM inhibitor KU-55933. DMSO, the solvent for Ku-99533, was used as negative control. Phosphorylation of SNEV and known ATM target γH2AX was reduced by inhibition of ATM. (**B**) Phopshorylation of SNEV at S149 is reduced in ATM conditional knockout MEF. Upper panel: wt and ATM -/- MEF were treated with 200 μM H_2_O_2_ for 1h, harvested, resuspended in 2x SDS loading dye and subjected to SDS PAGE. Western Blot was detected with anti-pSNEV(S149) and anti-βActin to ensure equael loading. Lower panel: Genomic DNA was isolated from wt and ATM lox/lox MEF before and after transfection with Cre and used in a genotyping PCR. The band for deleted ATM is present only after transfection with Cre, nonetheless the deletion is not quantitative and a small portion of ATM is maintained.

Indeed, we observed markedly reduced levels of phosphorylated SNEV upon oxidative stress, when ATM was blocked by Ku-55933. Similarly, upon oxidative stress we detected SNEV as a double band with anti-SNEV antibody, and Ku-55933 treatment abolished the upper band, supporting the idea that this upper band represents phosphoSNEV (Fig. 4A). The fact that the phosphoSNEV bands did not disappear completely could be due to another kinase that backs up ATM function. It is known that both ATM and ATR can phosphorylate H2AX [34], which also explains that H2AX phosphorylation is only slightly reduced upon ATM inhibition.

To confirm that ATM controls SNEV S149 phosphorylation with an independent method, we used mouse embryonic fibroblasts in which ATM was flanked by loxP sites and cut out using Cre recombinase (ATM -/- MEF). As shown in fig. 4B, the induction of pSNEV(S149) upon hydrogen peroxide treatment was reduced to less than half of that observed in wildtype MEF (pSNEV band intensity 0.16 versus 0.38 arbitrary units, normalized to βActin intensity; data not shown). The lower panel in fig. 4B shows an agarose gel of the genotyping PCR. Using a combination of three primers, three different fragments are amplified according which cells can be genotyped. The 320 bp fragment indicating absence of loxP sites is amplified only in wildtype cells, whereas presence of loxP sites gives rise to a 420 bp fragment. Only after introduction of Cre recombinase is ATM cut out and a fragment of 920 bp is amplified. Since the 420 bp fragment is still present inspite of Cre transfection, there is still residual ATM activity in our ATM -/- MEF, which explains why we still detect some pSNEV in these cells, although to a lesser extent then in wt MEF.

Taken together, this suggests that SNEV is phosphorylated upon DNA damage in an ATM-dependent manner and the presence of the ATM target motif suggest that it might be a novel direct substrate for ATM.

### Effect of phosphorylation on life span and stress resistance in HUVEC

**Figure 5:**
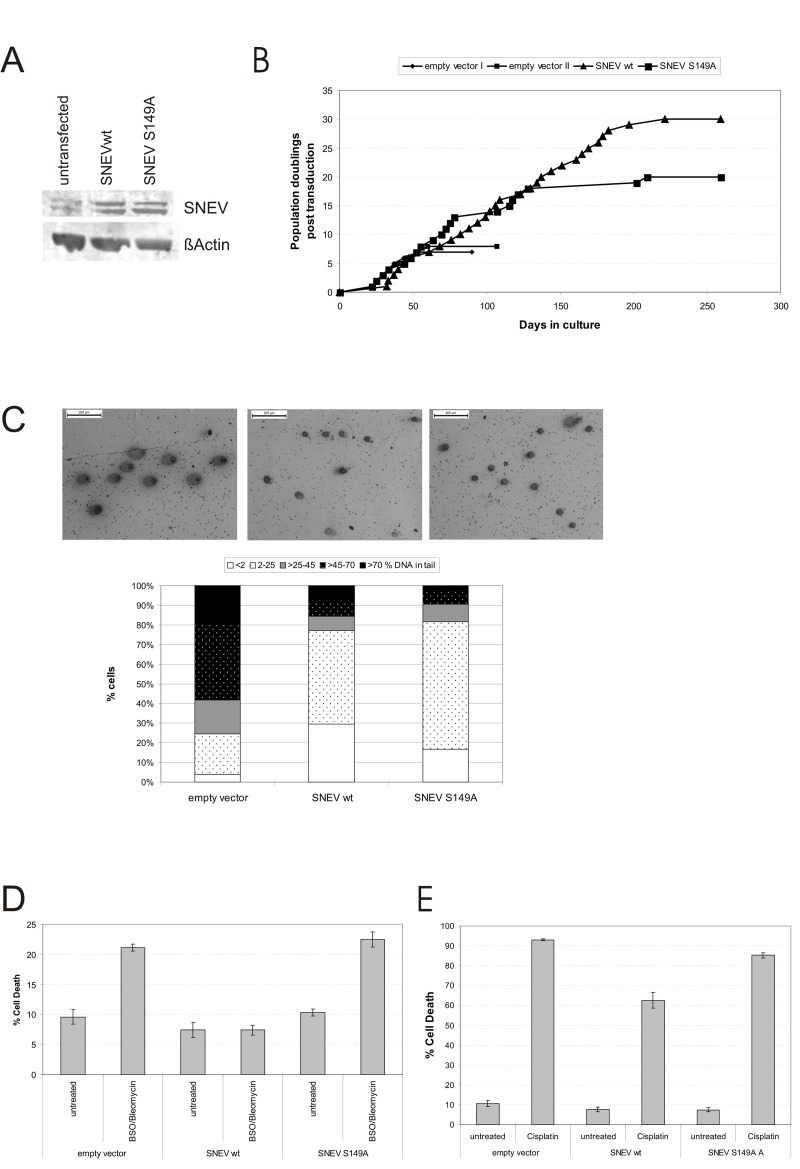
Phosphorylation at S149 is necessary for apoptosis resistance and partially for life span extension conferred by SNEV (**A**) Normal HUVEC as well as stable HUVEC overexpressing SNEV wt, SNEV S149A or empty vector control cells were lysed and submitted to SDS PAGE. Western Blots were probed with anti-SNEV and anti-?Actin antibodies. SNEV protein expression levels are increased five-fold in SNEV wt and ten-fold in SNEV S149A HUVEC as compared to untransfected HUVEC. (**B**) Growth curves of HUVEC overexpressing SNEV wt, SNEV S149A and empty vector. (**C**) SNEV wt as well as SNEV S149A overexpression reduces the basal level of DNA damage. Upper panel: Representative pictures of Comet assay performed with empty vector, SNEV wt and S149A point mutant overexpressing HUVEC. Lower panel: Comets were classified into 5 categories depending on the percentage of the DNA content in the tail. DNA damage levels of SNEV S149A. (**D**) Cells were pre-incubated with 1 mM BSO for 48h, followed by treatment with 100 μg/ml Bleomycin for 24h. Apoptosis was measured by Annexin-FITC and PI staining and subsequent flow cytometric analysis. (**E**) Cells were incubated with 100μg/ml Cisplatin for 24h. Apoptosis was measured as in **D**.

To investigate the physiological effect of the phosphorylation at S149 of SNEV, we generated stable HUVEC cell lines overexpressing either wildtype SNEV (wtSNEV HUVEC) or a point mutant SNEV where the ATM target site was abolished by exchanging serine 149 for alanine (SNEV S149A HUVEC). Both recombinant cell lines showed comparable overexpression of SNEV versus untransfected HUVEC (Fig. 5A). Overexpression was additionally confirmed by IF (Fig. S5).

Using these cell lines, we studied how the lack of phosphorylation at S149 affects DNA damage levels, stress resistance and life span.

As described earlier [26], overexpression of wtSNEV had a strong life-span extending effect. Indeed, SNEV wt HUVEC underwent 30 population doublings post transduction, while empty vector control HUVEC reached senescence already upon PD 8 post transduction, thus SNEVwt overexpression more than tripled the replicative life span. Surprisingly, SNEV S149A overexpression did not fully abrogate this effect of SNEV and also extended the replicative life span of HUVEC, albeit to a significantly lower extent of around 18 PD post transduction (Fig. 5B). Replicative senescence at the respective replication end points was confirmed by staining for the widely accepted senescence marker SA β-galactosidase expression (data not shown).

Since we bserved phosphorylation of SNEV upon treatment with DNA damaging agents, we hypothesized that phosphorylation might be necessary to elicit a proper DNA damage response. Therefore we performed comet assays to test for DNA integrity under basal conditions.

Indeed wtSNEV overexpressing cells were mainly assigned to categories I or II, corresponding to no or low levels of DNA damage. In contrast, the SNEV S149 HUVEC have lower levels of DNA damage than empty vector controls, but still increased damage levels compared to wtSNEV HUVEC. We conclude that increased SNEV levels lead to increased DNA maintenance in unstressed conditions, while the full effect of more efficient repair unfolds only if SNEV can be phosphorylated at S149.

In addition to the basal levels of DNA damage, we assessed the cellular response to treatment with genotoxic and/or oxidative stress inducing reagents. Therefore, we used combined BSO/bleomycin treatment as described earlier [26]. WtSNEV HUVEC did not enter apoptosis upon the treatment in accordance to our earlier results [26], which is also in agreement with findings by the Legerski lab, that GFP-SNEV overexpression in Hela cells reduces MMS induced apoptosis by two-fold [24]. In contrast, in SNEV S149A HUVEC the BSO/bleo-mycin treatment clearly induced apoptosis/necrosis. The initially low level –when compared to empty vector control cells- of cell death of 10% for SNEV S149A HUVEC more that doubled upon treatment to 22.5%.

Since SNEV is required for repair of DNA interstrand cross-links [25], we tested if SNEV overexpression protected cells from the deleterious effects of the DNA cross-linking agent cisplatin. Figure 5E shows that cisplatin treatment very strongly induced apoptosis in empty vector control HUVEC as well as in SNEV S149A HUVEC, while wtSNEV HUVEC were clearly more resistant to this treatment. Therefore, we conclude that phosphorylation of SNEV at S149 is necessary for efficient corsslink-repair activity of SNEV.

In summary, we conclude that overexpression of S149-phosphorylation incompetent SNEV does have a small but discernible effect on the DNA damage status under normal culture conditions when compared to wtSNEV overexpression, but does not exert the strong anti-apoptotic effect of wtSNEV after induction of DNA damage by either oxidative stress or interstrans crosslinks.

In any case, the life-span extending and DNA repair functions of SNEV do partially depend on phosphor-rylation at S149, most probably in dependence of ATM.

## DISCUSSION

Here, we present SNEV as a novel ATM-dependent phosphoryalation substrate in response to oxidative stress induced DNA damage. We show that SNEV is phosphorylated at S149 in an ATM-dependent manner in different cell types in response to various DNA damage. Surprisingly, ATM-dependent phosphorylation at S149 is essential for suppressing oxidative stress induced apoptosis, but dispensable for efficient DNA break repair and accounts for part of the cellular life span extension observed upon SNEV overexpression.

A plethora of mutations in the DNA damage response pathways, specifically in transcription-coupled nucleotide excision repair (TC-NER) is known to be responsible for premature ageing syndromes in humans (reviewed in [35]) Accordingly, gene knock-outs of these factors also show premature aging in mice. However, to the best of our knowledge, only two studies have so far reported that DNA repair factors upon overexpression extend the life span of model systems. One example is overexpression of the repair factor GADD45 in the nervous system of D. melanogaster that increased the life span of these animals [36], while overexpression in the whole animal was lethal [37]. The other example is mice with single extra copies of the tumor suppressor p53 and its stabilizer Arf, which led to an extension of the median life span by 16% compared to controls, while the maximum life span was not increased [38].

We have recently observed that a multi-talented protein, SNEV, extends the replicative life span of endothelial cells upon overexpression, reduces basal levels of DNA damage and suppresses oxidative stress induced apoptosis in these cells [39]. The multiple talents of SNEV, however, include a well documented function in DNA repair [23-25,40], but also in pre-mRNA splicing, ubiquitination and proteasomal breakdown as well as adipogenic and neurogenic differentiation, where another phosphorylation sites plays a crucial role [28,29]. Therefore, it is not yet clear which function of SNEV is responsible for the life span extending activity.

We here identified an ATM consensus phosphorylation site on SNEV. Our experimental design does not provide direct evidence that ATM phosphorylates SNEV at this site. However, SNEV's highly conserved yeast homologue Prp19 is a direct phosphorylation target of Tel1 and/or Mec1, the yeast homologues of ATR and ATM [41], making it more likely that also in humans this direct link is conserved.

Since ATM dependent phosphorylations are key signals of DNA damage and oxidative stress, we hypothesized that ATM dependent phosphorylation of SNEV might contribute to cellular life span extension as consequence of activating SNEV's DNA repair activity. Therefore, we overexpressed the phosphorylation-incompetent point mutant versus wild type SNEV and found that the DNA damage status of cells in unstressed conditions overexpression is slightly but not dramatically reduced by the point mutation. Surprisingly, suppression of apoptosis upon oxidative stress treatment as seen upon SNEV overexpression was completely abolished by S149A mutation, suggesting an important role of ATM dependent phosphorylation of SNEV for induction of apoptosis. It is tempting to speculate that at least part of this high susceptibility of ATM deficient cells to apoptosis [13,42,43] is dependent on the inability to phosphorylate SNEV. Furthermore, the susceptibility of ATM deficient cells to undergo apoptosis is not only dependent on DNA damaging agents, but also on oxidative stress [44], well in accordance with our data.

How might the S149 phosphorylatable form of SNEV suppress apoptosis? Phosphorylated SNEV localizes under basal conditions exclusively to the cytoplasm, but is detected predominantly in the nucleus after DNA damage. Although it is likely that ATM phosphorylation occurs in the nucleus, it cannot be ruled out that phosphoSNEV is imported into the nucleus. This would be supported by the notion that amino acids 1-166 of mouse SNEV are necessary for nuclear import [45] and the phosphorylation site lies within this portion possibly conferring a nuclear import signal. However, one report suggests that cytoplasmic SNEV is responsible to suppress apoptosis, at least under conditions of hypoxia. Under hypoxic conditions, a particular form of redox stress, SNEV is observed to interact with PHD3 in the cytoplasm [46], an interaction that suppresses cell death. In addition, GFP-SNEV, which locates preferentially in the cytoplasm (compared to untagged SNEV), has a stronger anti-apoptotic effect than untagged SNEV, indicating the cytoplasmic localization of SNEV is important for its anti-apoptotic role. We find that upon overexpression, the cytoplasmic portion of SNEV is increased when compared to untransfected HUVEC (See Fig. S3), backing up the importance of cytoplasmic SNEV in preventing apoptosis. On the other hand, also in SNEV S149A HUVEC, the ratio of nuclear versus cytoplasmic SNEV is shifted towards the cytoplasm in a manner similar to SNEV wt HUVEC, but still these cells undergo apotosis upon oxidative stress, indicating that the phosphorylatability of cytoplasmic SNEV is essential for its anti-apoptotic effect. Under normoxic conditions, SNEV interacts with PHD3 via its C-terminus, while under hypoxic conditions, a hypoxia inducible interaction domain comprising amino acids 66-220 stabilizes this interaction [46]. Since this region covers the S149, it is tempting to speculate that ATM-dependent phosphorylation at S149 is the signal for increased binding to PHD3, leading to apoptosis suppression, while the non-phosphorylatable S149A mutant binds PHD3 with lower affinity, allowing for caspase activation by PHD3. It is also unclear, how the phosphoSNEV would accumulate in the cytoplasm, as it is not detected there upon stress with our antibody. It will be interesting to see if a phospho-mimicking S149D point mutant further increases the anti-apoptotic effect of SNEV overexpression and where it will localize.

The finding that the point mutant only slightly improves the basal DNA damage status of the cells as visualized by comet assays suggests that the here identified phosphorylation site might be decoupling the DNA repair activity from the apoptosis signalling activity of SNEV.

It remains to be elucidated how this translates into the extended life span of SNEV overexpressing cells. Suppression of apoptosis might reduce the loss of cells during cultivation and thus increase the total replicative life span merely by reducing the replicative exhaustion of the cells. However, the apoptosis competent cells of the S149A mutant overexpression are still markedly longer lived than empty vector controls. This would suggest that the full life span extension as observed by wtSNEV overexpression depends on both, apoptosis suppression and the lower basal DNA damage of the cells and thus on SNEV's DNA repair activity.

Taken together, our data support the hypothesis that DNA damage repair and signalling factors can extend the life span of model systems, as in this case the replicative life span of endothelial cels and thus support the idea that a genetically programmed DNA repair ability counteracts and slows down the stochastic accumulation of DNA damage and thus the aging process.

## METHODS

### Cells and culture conditions

HeLa cells were grown in RPMI1640 (Biochrom AG, Berlin, Germany) supplemented with 10% fetal calf serum (FCS).

Human diploid fibroblasts (HDF) were grown in DMEM/HAM's F12 1:2 (Biochrom AG, Berlin, Germany) supplemented with 10%FCS.

Human umbilical vein endothelial cells (HUVEC) were cultivated in EBM Basal Medium plus EGM SingleQuot Supplements & Growth Factors (Lonza, Basel, Switzerland) supplemented with FCS to a final 10%. For HUVEC, flasks were precoated with 0.1% gelatine in PBS.

GFP-SNEV-Hela were established by transfecting Hela cells with a SNEV-BAC construct containing all regulartory elements obtained from Ina Poser, Max Planck Institute for Molecular Cell Biology and Genetics, Dresden, Germany. GFP-SNEV-Hela were cultivated in RPMI1640 (Biochrom AG, Berlin, Germany) supplemented with 10% fetal calf serum (FCS) and 800 μg/ml G418 (Gibco, now life technologies, Grand Island, NY, USA).

Confluent adherent cultures were detached using 0.1% trypsin and 0.02% EDTA and were passaged with an appropriate split ratio of 1:2 or 1:4 once or twice a week depending on confluence and population doubling level (PD). Subsequently, cumulative PDs were calculated as a function of passage number and split ratio [47].

Fibroblasts (HDF5) 2x10^5^ /well were seeded in 6 well plates. The next day, cells were treated with H_2_O_2_ (100, 200, 400 μM/ml) for 1 hour and 100μM/ml for different time spans (5, 15, 30, 60, 120 minutes).

Fibroblasts (HDF5) were seeded in 6 well plate at a density of 2x10^5^ cells/well 24 hours before treatment with 2.5, 5, 10, 25, 50, 100μg/ml of Bleomycin for 1 hour and 25μg/ml for (5,15,30,60,120 minutes). For Mitomycin C treatment, fibroblasts cells were exposed to different concentration (2.5, 5, 10, 25, 50 μg/ml) for 24 hours. Cell were scraped directly on ice in 2x SDS loading dye, sonicated and boiled. Proteins were separated by SDS gel electrophoresis in 4-12% BT gel (Invitrogen, now life technologies, Grand Island, NY, USA).

To induce apoptosis, cells were seeded into 12-well cell culture plates at a density of 10000 cells /cm^2^. To enhance the sensitivity of cells to bleomycin-induced apoptosis, cells were cultivated in the presence of 1 mM buthionine-sulfoximine (BSO, Sigma-Aldrich, St.Louis, MO, USA) for 48 h before treatment with with 100 μg/ml of bleomycin (Sigma-Aldrich, St.Louis, MO, USA) for further 24 h. Alternatively, cells were treated with 100 μg/ml of cisplatin (Sigma) for 24h to asssay apoptosis induction by DNA cross-links.

### ATM deletion in mouse embryonic fibroblasts

Primary mouse embryonic fibroblasts (MEF) were prepared from E13.5 fetuses (*ATM^flox/flox^*). Cells were cultured in DMEM high glucose medium (Sigma-Aldrich) supplemented with 15% fetal bovine serum (Lonza), 1% L-glutamine (Gibco, now life technologies, Grand Island, NY, USA), 1% penicillin and strepromycin, 1% NEAA (non essential amino acids, Gibco), 1% sodium pyruvate (Gibco) and β-mercaptoethanol (1:140000).

Large T-Antigen was used to immortalize MEFs (*ATM^flox/flox^*). For the generation of retrovirus, Large T-Antigen -MSCVneo plasmid was co-transfected with the packaging plasmid pCL-Eco and the enveloping plasmid VSV-G in 293T cells. Supernatant was collected after 24 and 48 hours. MEFs were transduced using 5 ml of filtered supernatant added to 5 ml cell culture medium in the presence of 8 mg/ml polybrene (Sigma-Aldrich). After 24 hours virus was removed. 14 days selection was started 72 hours after infection using 400μg/ml neomycin (Sigma-Aldrich).

In a second infection step, Large T-Antigen-immortalized MEFs were transduced with the Cre-pBabe-puro retrovirus for the Cre-mediated deletion of *ATM*. Selection was started 72 hours after transduction with 2 μg/ml puromycin for 7 days. Medium was exchanged every Deletion of ATM was verified by PCR of genomic DNA using primer 1 (5' ATC AAA TGT AAA GGC GGC TTC 3'), primer 2 (5'CAT CCT TTA ATG TGC CTC CCT TCG CC 3') and primer 3 (5'GCC CAT CCC GTC CAC AAT ATC TCT GC 3') giving rise to three fragments representing absence or presence of loxP sites or deletion of ATM.

### Plasmid construction, generation of recombinant retroviruses and cell line establishment

SNEV cDNA was amplified by PCR and ligated into the retroviral plasmid pLenti6. The SNEV S149A point mutant was generated using the QuikChange Multi Site-Directed Mutagenesis Kit (Agilent, Santa Clara, CA, USA). The negative control vector contained only the blasticidin resistance gene. Retroviral particles were generated according to the manufacturer's protocol (Invitrogen). For cell line establishment, HUVECs (PD14) were infected with retroviral particles according to the manufacturer's protocol (Invitrogen, now life technologies, Grand Island, NY, USA) at a multiplicity of infection (MOI) of 4 in EBM with 10% FCS supplemented with 8 μg/ml Polybrene. Thereafter, transfectants were selected using 5 μg/ml Blasticidin. Arising cell clones (similar number in all experiments) were grown as mass culture. PDs post transfection (pT) were calculated starting with the first passage after selection was completed.

### Antibodies

Prp19/Pso4 rabbit polyclonal antibody was from Bethyl Laboratories (Montgomery, TX, USA) #A300-102A. ATM antibody [2C1] mouse monoclonal antibody was from Gene Tex (Irvina, CA, USA) #GTX70103. anti-pSNEV(S149) rabbit polyclonal antibody was generated by Moravian Biotechnology Ltd. (Bratislava, Slovakia). Gamma H2A.X (phospho S139) mouse monoclonal antibody [9F3] was from Abcam (Cambridge, UK) #26350. β-Actin mouse monoclonal antibody was from Sigma-Aldrich (St.Louis, MO, USA) #A-5441. GAPDH rabbit antibody FL-335 was from Santa Cruz (Santa Cruz, CA, USA) #sc-25778.

### SDS PAGE and Western Blotting

For SDS PAGE, protein samples were mixed with 4x SDS loading dye (240 mTris-Cl pH 6.8, 8% SDS, 40% glycerol, 0.05% bromophenol blue, 5% β-Mercaptoethanol) and heated to 75°C for 10min. Samples were separated on a NuPAGE 4-12% Bis/Tris polyacrylamide gel (Invitrogen, Carlsbad, CA; USA) in MOPS buffer at 200V. Electrophoresis and blotting to PVDF membrane (Roth, Karlsruhe, Germany) were performed using the XCell *SureLock*®Mini-Cell (Invitrogen, now life technologies, Grand Island, NY, USA) in accordance to the manufacturer's protocol. After incubating with blocking buffer (3% skim milk powder in PBS with 0.1% Tween-20) on an orbital for 1h at room temperature or overnight at 4°C, the membranes were incubated for 1h or overnight with the primary antibody diluted in blocking buffer, followed by 1h incubation with secondary anti-Rabbit-IR-Dye 800 and/or anti-Mouse-Alexa 680 (Licor), both diluted 1:10000 in blocking buffer. Both antibody incubations were followed by 3 washes with PBS with 0.1% Tween-20. Membranes were scanned using the Odyssey infrared imaging system (LI-COR, Lincoln, NE, USA). Mouse anti-?Actin (Sigma-Aldrich, St.Louis, MO, USA) or rabbit anti-GAPDH (Santa Cruz, Santa Cruz, CA, USA) were used as loading controls.

### Dephosphorylation

For dephosphorylation experi-ments, H_2_O_2_ treated or untreated HeLa cells were harvested by trypsinization, collected by centrifugation at 170g for 10 min, washed twice with PBS and resuspended in NEB3 Buffer (New England Biolabs, Ipswich, MA, USA), in which Calf intestinal phosphatase (CIP; New England Biolabs, Ipswich, MA, USA) has optimal activity. Cells were lysed by sonication and subsequent centrifugation at maximum speed for 30 min at 4°C. Cleared lysates were split and one half was incubated with 10U CIP for 30 min at 37°C, whereas the other half was mock treated. Subsequently, samples were submitted to Western Blot analysis.

### Anti-GFP Trap IP

GFP-SNEV-Hela were grown to 90% confluence in T175 roux flask and incubated with 100 μM H_2_O_2_ to induce SNEV phosphorylation or left untreated as negative control. Cells were scraped on ice in 3ml Lysis buffer (50 mM Hepes-KOH pH 7.5, 5 mM EDTA pH8, 150 mM KCl, 10% glycerol, 1% Triton X-100, 20 mM β-glycerophosphate, 10mM Na-pyrophosphate, 10 mM NaF, 1 mM DTT, 1 mM Na_3_VO_4_, 0.1mM PMSF, 20 μg/ml Leupeptin, 20 μg/ml Chymostatin, 20 μg/ml Pepstatin, 1μM Okadaic acid) per T175 and sonicated using 3 bursts of 10s. Lysate was cleared by centrifugation for 30 min at full speed. All centrifugation stepy were carried out at 4°C. 30 μl GFP-Trap Beads (ChromoTek, Munich, Germany) were equilibrated by washing thrice in 500 μl ice cold wash buffer (50mM Hepes-KOH pH 7.5, 5mM EDTA pH8, 150mM KCl, 10% glycerol, 0.05% NP-40, 20 mM β-glycerophosphate, 10 mM Na-pyrophosphate, 10mM NaF, 20 μg/ml Leupeptin, 20 μg/ml Chymostatin, 20 μg/ml Pepstatin) and collected by centrifugation at 2,700 x g for 2 min. 1 ml Lysate corresponding to 1 mg total protein was added to the beads mixed on an overhead shaker for 1h at 4°C. Beads were collected by centrifugation for 2 minutes at 2,000 x g. Discard supernatant and wash beads 5x times with 500μL ice cold wash buffer (ocadaic acid added only for the first two washing steps) and finally resuspended in washing buffer.

### Mass spectrometric analysis

Beads were washed five times with ammonium bicarbonate buffer (50mM ABC). Disulfide bonds were reduced by incubation with dithiothreitol (DTT, 5% w/w of the estimated amount of protein) for 30 min at 56°C and Cys-residues were subsequently alkylated with iododacetamide (IAA, 25% w/w of the estimated amount of protein) for 20 min at RT in the dark. DTT (2.5% w/w of the estimated amount of protein) was added to consume excess IAA and proteins were digested with subtilisin for 1 hour at 37°C. Digests were stopped by addition of trifluoro acetic acid (TFA) to approx. pH 3. 10% of the peptide mixture was analysed directly, the remaining 90% were enriched for phosphopeptides by the use of TiO2 as described in [48]. Peptides were separated on a U3000-HPLC-system (Dionex, Sunnyvale, CA, USA). Peptides were loaded on to a the trapping column (PepMAP C18, 0.3 × 5 mm, Dionex) with 0.1% TFA as loading solvent and then eluted onto an analytical column (PepMAP C18, 75 μm × 150 mm, Dionex) with a flow rate of 300 nl/min and a gradient from 0% solvent B to 100% solvent B in 90min, followed by a washing step of 10 min with 10% solvent B and 90% solvent C (solvent B: 40% acetonitril (ANC), 0.08% formic acid, solvent C: 80% ACN, 10% trifluorethanol, 0.08% formic acid). The HPLC system is online coupled to a Velos Orbitrap mass spectrometer (Thermo Scientific, Waltham, MA, USA) equipped with an ESI-source (Proxeon, now Thermo Scientific). A full scan (scan range 400-1800 Th, resolution 60.000) was followed by MS2 analysis by collision-induced dissociation (CID) of the 20 most intense precursors in the linear iontrap. Normalized collision energy was set to 35%, activation Q at 25 and activation time at 30 ms. Peptide identification was performed using the SEQUEST algorithm in the ProteomeDiscoverer 1.3.0.339 software package (Thermo Scientific). Carbamidomethylation of Cys was set as static modifications, phosphorylation of Ser/Thr/Tyr and oxidation of Met were set as variable modifications. Spectra were searched against a small database plus contaminants for a fast PTM analysis. Search parameters were no protease specificity, a peptide tolerance of 2 ppm, a fragment ions tolerance of 0.8 Da. The results were filtered at the XCorr values to an FDR of 1% on the peptide level. The probability of phosphosite localization was calculated using the phosphoRS 2.0 software [49] implemented into the Proteome Discoverer.

### Indirect immunofuorescence staining

Cells were seeded on coverslips one day prior to immunofluorescence staining. The next day, cells were washed with PBS and fixed with 3.7% (w/v) for 20 min at room temperature. Permeabilizationwas performed with 0.1% Triton X-100 in PBS for 10 min at room temperature. Cells were incubated with primary antibodies diluted in PBS with10% FCS for 1 h, washed 3 times for 10 min withPBS, incubated for 1 h with the appropriate secondaryantibodies diluted in PBS with 10% FCS, and washed 3 timesfor 10 min with PBS. Anti-pSNEV (S149) was diluted 1:250. As secondary antibodies, anti mouse dyelight488 1:1000 and dyelight649 1:1000anti-rabbit or anti-mouse antibodies were used. Microscope as described previously.

To visualize the nuclei, cells were counterstained with 4',6-diamidino-2-phenylindole (DAPI). Cells were washed and fixed as above and subsequently incubated with 100ng/ml DAPI in PBS for 10 min at room temperature.

After staining, slides were mounted on cover slips using slow fade gold Mounting Medium for Fluorescence (life technologie, Grand Island, NY, USA) and sealed with nail polish.

Microscopyand image analysis were carried out using a Leica SP5 II laser scanning confocal microscope (Leica Microsystems CMS, Mannheim, Germany).

### Comet assay

Single-and double-stranded DNA damage in SNEVwt and SNEV S149A overexpressing HUVEC under basal conditions was measured by single-cell gel electrophoresis (comet assay) under alkaline conditions as described previously [50]. At least 100 randomly selected cells per slide were examined for the presence or absence of comets using fluorescence microscopy. The cells were assigned to five different categories according to their DNA content in tail using the TriTek CometScore software. Percentage of cells in each category was calculated.

### Apoptosis staining

Cells were detached using 0.1% Trypsin/0,02%EDTA and stained with Annexin V-Bacific Blue (Invitrogen, now life technologies) and PI (Roche, Basel, Switzerland) according to the manufacturer's instructions. Analysis of the percentages of apoptotic and necrotic/late-apoptotic cells was performed using a BD FACS Canto II flow cytometer (Becton Dickinson, Franklin Lakes, NJ, USA) and the FCS Express V3 software (De Novo Software, Los Angeles, CA, USA).

## SUPPLEMENTAL FIGURES AND TABLE

Fig. S1

Fig. S2

Fig. S3

Fig. S4

Fig. S5

Supplemental Table
