# Supplementary figures and images for: ATM-dependent phosphorylation of SNEVhPrp19/hPso4 is involved in extending cellular life span and suppression of apoptosis
Source: Aging (Albany NY). 2012 Apr 20;4(4):290–304. doi: 10.18632/aging.100452 (PMC3371764; doi:10.18632/aging.100452)

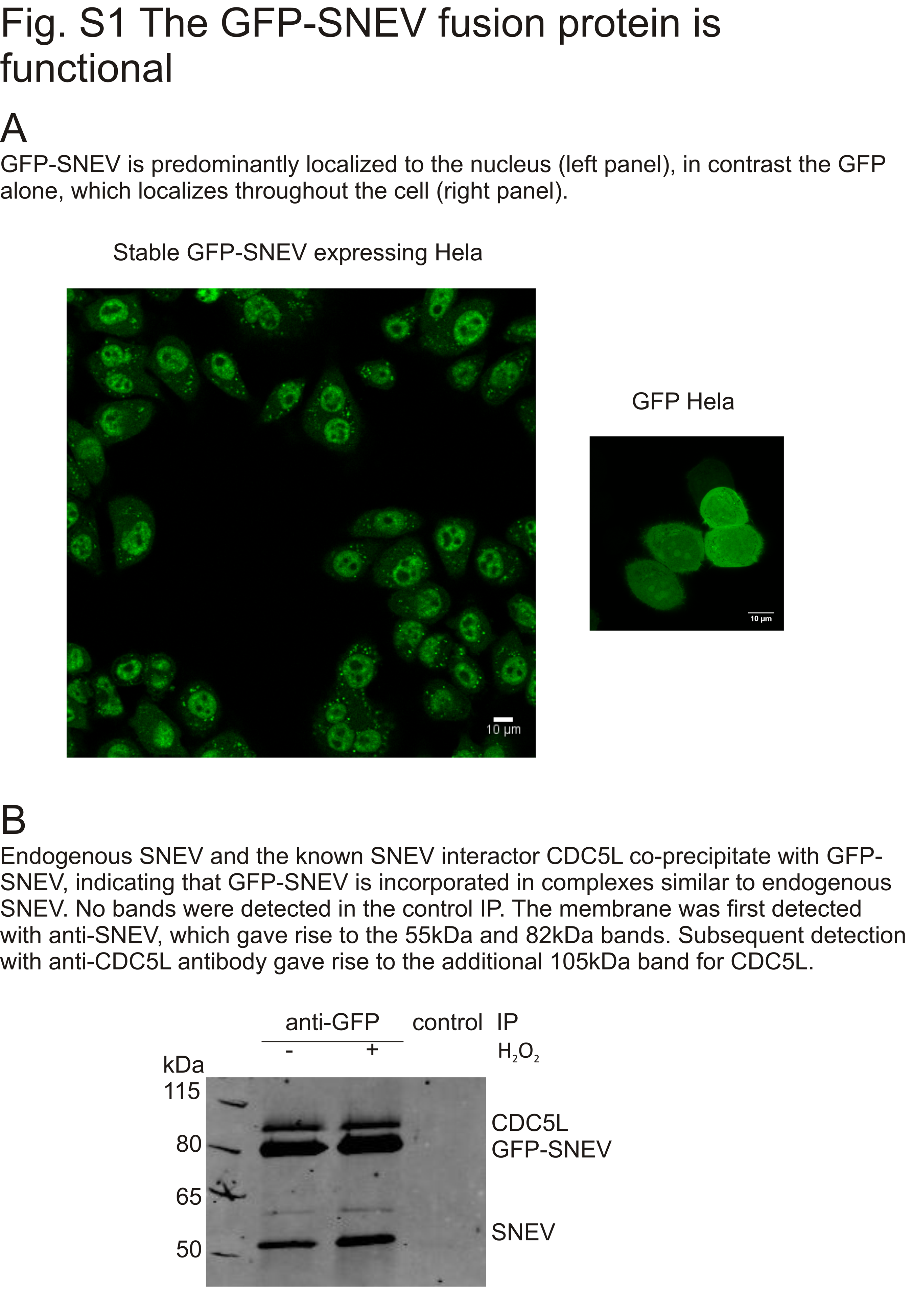

Supplement: Fig. S1 [file aging-04-290-s001.tif]

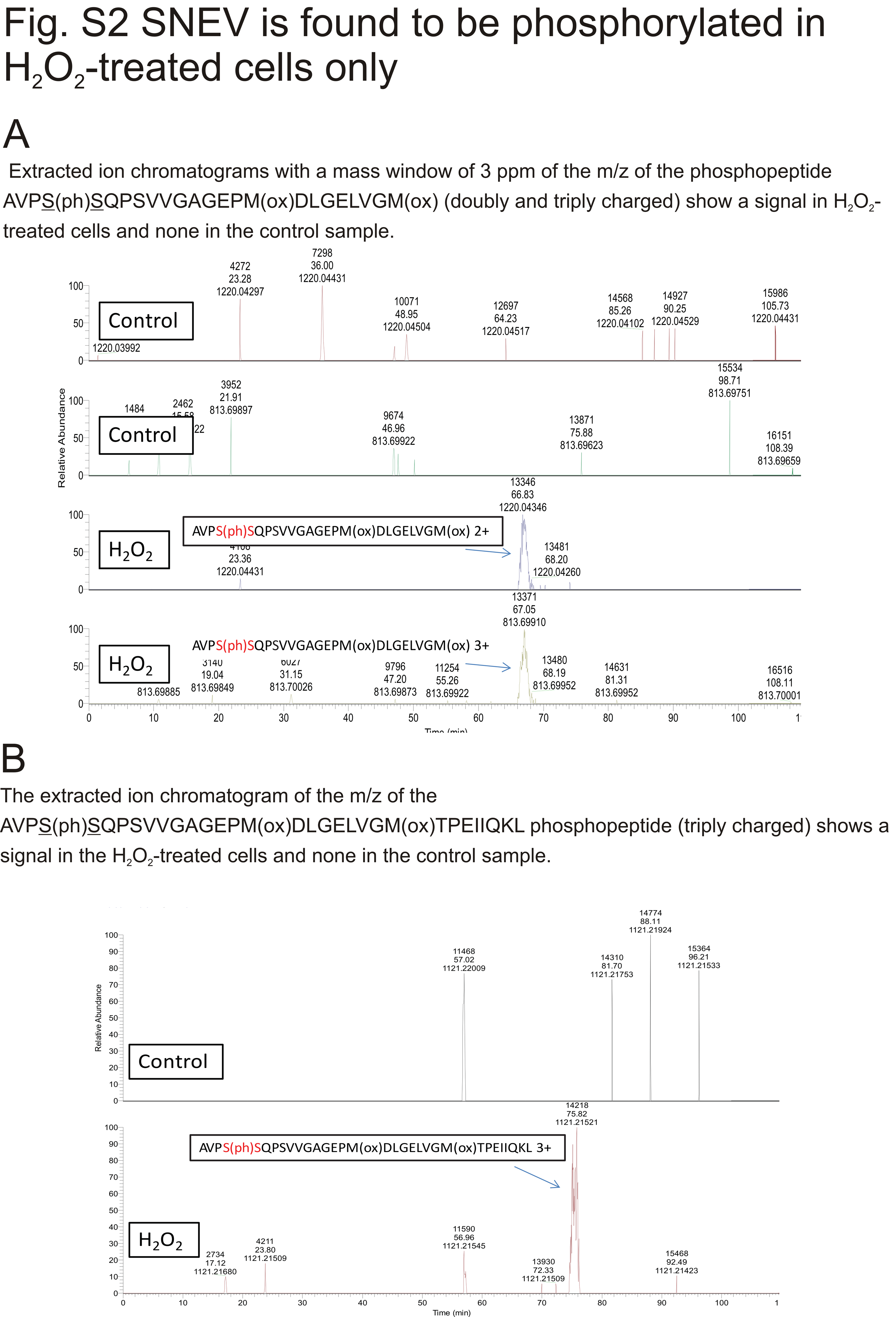

Supplement: Fig. S2 [file aging-04-290-s002.tif]

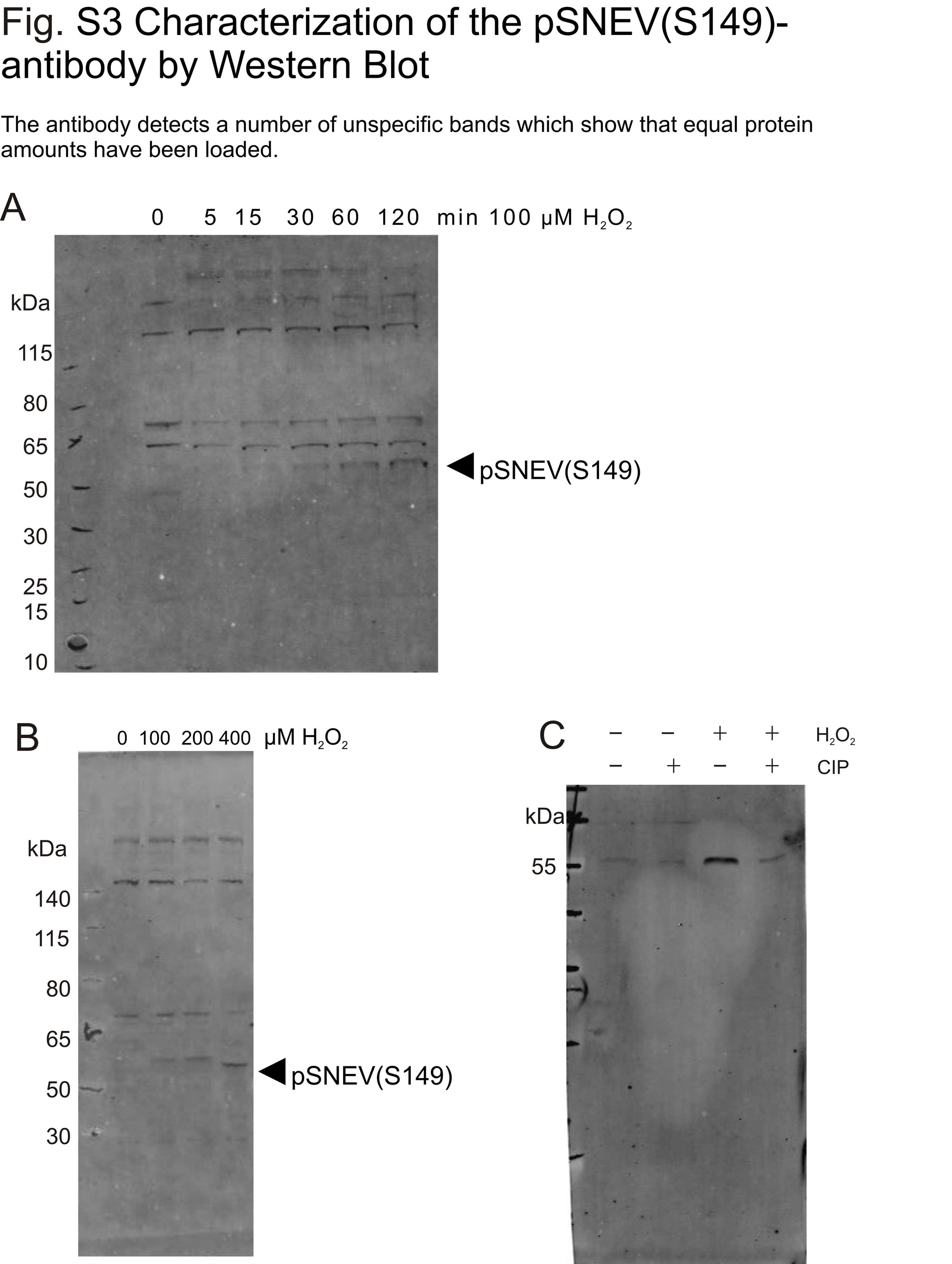

Supplement: Fig. S3 [file aging-04-290-s003.tif]

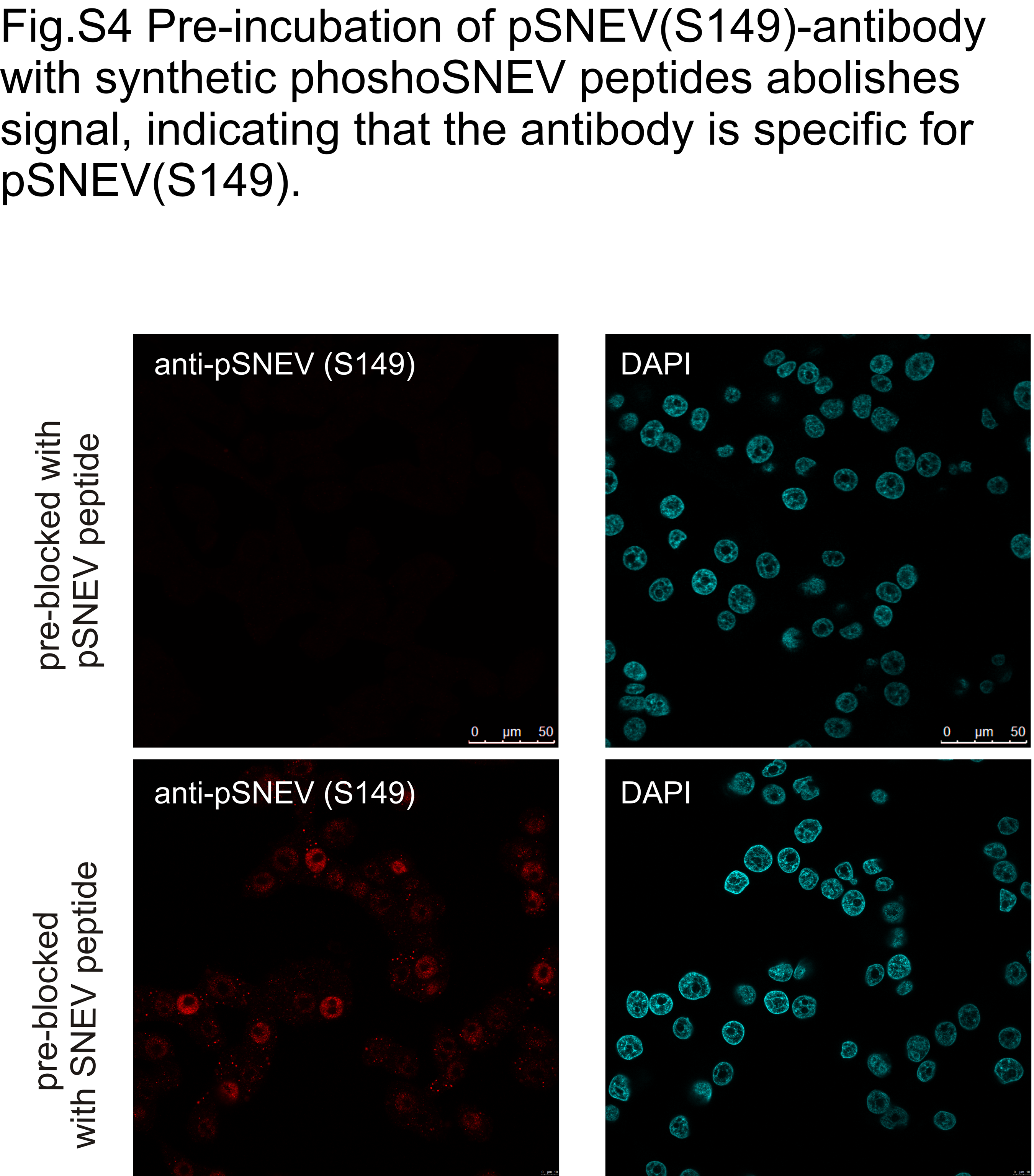

Supplement: Fig. S4 [file aging-04-290-s004.tif]

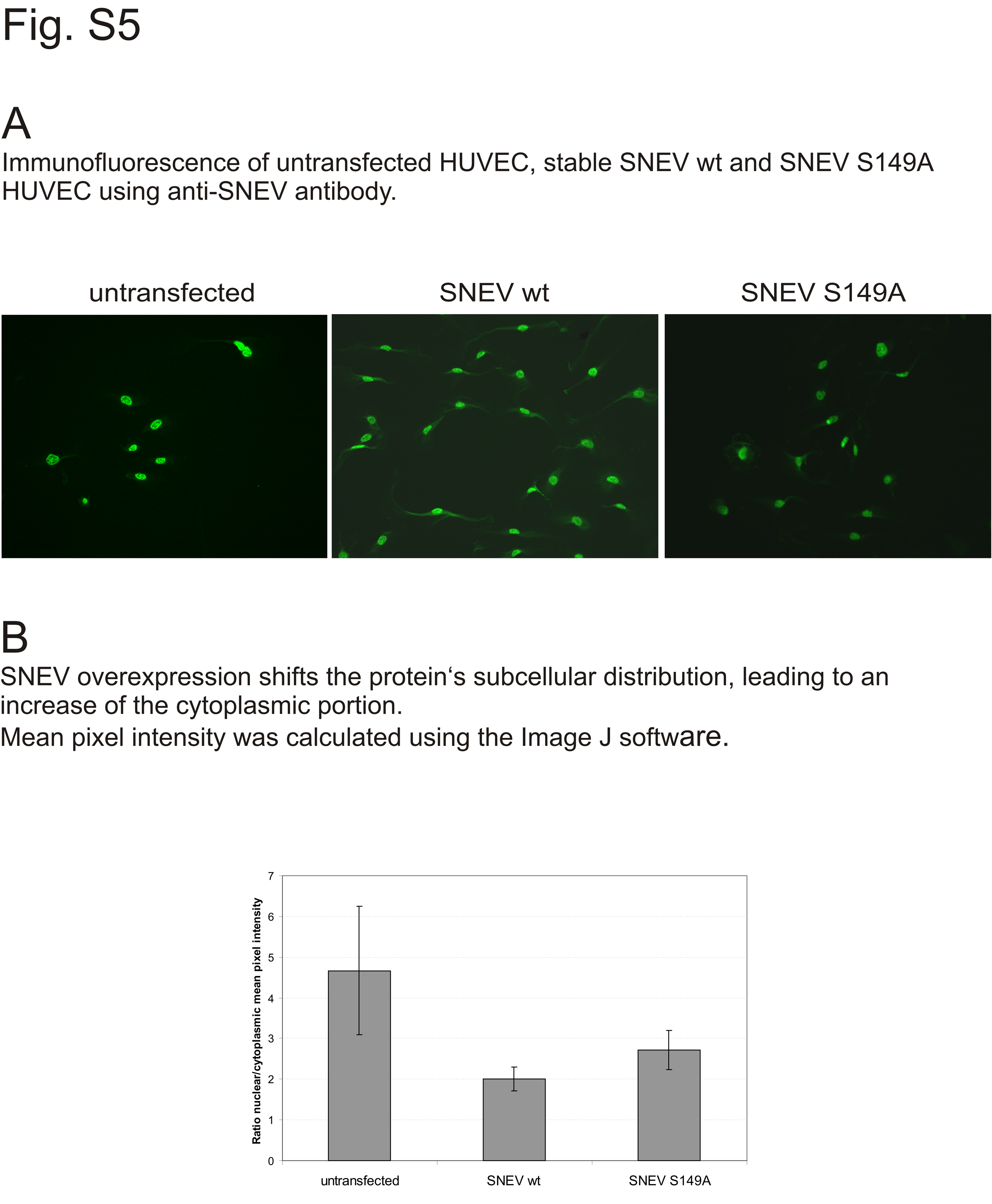

Supplement: Fig. S5 [file aging-04-290-s005.tif]
